# Progress in Hematopoietic Stem Cell Transplantation and Cellular Therapies

**DOI:** 10.3390/jcm11247354

**Published:** 2022-12-10

**Authors:** Diana Cenariu, Horia Bumbea, Anca Colita, Catalin Constantinescu, Minodora Desmirean, Sabina Iluta, Daniel Lysák, Alberto Mussetti, Ioana Tichil, Alina Tanase, Ciprian Tomuleasa

**Affiliations:** 1 Medfuture Research Center for Advanced Medicine, Iuliu Hatieganu University of Medicine and Pharmacy, 400337 Cluj-Napoca, Romania; 2 Department of Stem Cell Transplantation, University Emergency Hospital Bucharest, 022314 Bucharest, Romania; 3 Department of Hematology, Carol Davila University of Medicine and Pharmacy, 022314 Bucharest, Romania; 4 Department of Stem Cell Transplantation, Fundeni Clinical Institute, 022328 Bucharest, Romania; 5 Intensive Care Unit, Emergency Hospital, 400006 Cluj-Napoca, Romania; 6 Department of Anesthesia and Intensive Care, Iuliu Hatieganu University of Medicine and Pharmacy, 400349 Cluj-Napoca, Romania; 7 Department of Hematology, Iuliu Hatieganu University of Medicine and Pharmacy, 400349 Cluj-Napoca, Romania; 8 Department of Hematology, Ion Chiricuta Clinical Cancer Center, 400124 Cluj Napoca, Romania; 9 Department of Hematology and Oncology, University Hospital Pilsen, 32300 Pilsen, Czech Republic; 10 Faculty of Medicine in Pilsen, Charles University, 32300 Pilsen, Czech Republic; 11 Clinical Hematology Department, Institut Català d’Oncologia-Hospitalet, 08901 Barcelona, Spain; 12 Institut d’Investigació Biomèdica de Bellvitge (IDIBELL), 08908 Barcelona, Spain

Hematological malignancies are considered to be one of the most important causes of mortality and morbidity in the modern world. Still, important progress has been made in the last decade due to breakthroughs in hematopoietic stem cell transplantation and cellular therapies. The results of these recent studies show that a promising road toward state-of-the-art treatment for the hematology patient, turning the diagnosis of leukemia or lymphoma into a curable disease, has already begun, with recent advances in hematopoietic stem cell transplantation and immunotherapy, and is now progressing forward. The prospect of providing a definitive alternative therapy for various malignancies, rather than conventional chemotherapy management, is indeed clinically, socially, and economically attractive, due to the huge medical, social, and economic burden that a malignancy imposes. This joint Special Issue presents modern approaches in the recent advances in stem cell transplantation for the hematological patient, with a special emphasis on progress in conditioning chemotherapy and cellular therapies. The published manuscripts address healthcare professionals that diagnose, treat, and research stem cell transplantation, with a special emphasis on state-of-the-art therapeutics and novel, emerging drugs and novel cellular therapies. Comprehensive reviews, original research (which may be basic or clinical research), or even interesting case series are presented, with the goal of presenting new approaches in the clinical management of patients diagnosed with a malignancy and undergoing stem cell transplantation, as well as approaches that may involve newly approved drugs, clinical trials in the pipeline, or even promising future therapeutic alternatives.

In Bucharest, Bumbea et al. showed that acute myeloid leukemia (AML) patients present changes in adhesion receptors and activation markers, suggesting a functional defect or denatured intracellular signaling in platelets [1]. The exposed data indicate that flow cytometry is able to effectively identify multiple functional platelet impairments in AML pathogenesis [1]. The Modified Early Warning Score (MEWS) is used worldwide as a track and trigger system that can help to identify patients at risk of critical illness. Calculated MEWS values considered the status at discharge (*p* < 0.0001) in the work of Constantinescu et al. [2], which assessed the frequency of death by MEWS. The authors calculated the hazard ratio (HR) of death depending on the selected MEWS cutoff. The best cutoff point was found to be ≥6, with an accuracy of 0.667, sensitivity of 0.675, specificity of 0.646, and AUC of 0.731. Patients with higher MEWS had a higher probability of mortality. Autologous stem cell transplantation is indicated for malignant lymphomas in the relapsed/refractory setting. Deak-Mihaly and Iluta et al. show that data is indeed limited and that results should be confirmed by multicentric clinical trials and should be regarded as single-center case series, despite their limitations [3]. Still, it offers a new therapeutic option for this rare subtype of malignant lymphomas, which has a dismal prognosis if left untreated [3].

In Pilsen, Lysak et al. proved that preoperative autologous blood donation is unnecessary for healthy marrow donors and could be indicated individually after considering the pre-collection hemoglobin level, donor and recipient weight, and expected blood loss. Reasonable substitution cutoffs have to be placed together with clinical symptom evaluation. The effective use thereof also requires an adequate time interval between PAD and BM harvest [4]. Transfusion medicine is not only of key importance in transplant medicine but also in plastic surgery, as demonstrated by a study carried out at the Victorian Adult Burns Service, which shows that transfusion trigger levels in stable patients may be amenable to review and reduction. Adjusted risk analysis can support the implementation of blood transfusion as a useful quality indicator in burn care [5].

As with all Special Issues, clinical the data stand on excellent theoretical background and reviews are especially important. Thus, Mussetti et al. emphasize the overall outcomes of post-transplant cyclophosphamide-based grafts versus host disease prophylaxis in the haploidentical setting and summarize the results obtained in the HLA-identical field. We present future perspectives at the end of the manuscript [6]. These data are backed up by hematopoiesis, clearly presented in the two cited reviews of Cenariu et al. and Desmirean et al. [7,8].
